# RNA interference-mediated gene silencing in murine T cells: *in vitro *and *in vivo *validation of proinflammatory target genes

**DOI:** 10.1186/1478-811X-6-3

**Published:** 2008-08-06

**Authors:** Tatjana C Gust, Luisa Neubrandt, Claudia Merz, Khusru Asadullah, Ulrich Zügel, Arne von Bonin

**Affiliations:** 1 Common Mechanism Research, Bayer Schering Pharma AG, Muellerstrasse 178, 13342, Berlin, Germany; 2 Target Discovery, Global Drug Discovery, Bayer Schering Pharma AG, Muellerstrasse 178, 13342, Berlin, Germany

## Abstract

**Background:**

T cells play a central role in many inflammatory diseases, hence the identification and validation of T cell-specific target genes will increase the understanding of T cell function in pathologic inflammatory situations. RNA interference (RNAi), with its ability to induce specific gene silencing in mammalian cells, represents a powerful technology to investigate and validate the function of pharmaceutical target genes *in vitro *and *in vivo*. The aim of the present study was to systematically explore RNAi-mediated gene-silencing of known T cell-specific model signaling molecules in primary murine T cells *in vitro *and *in vivo*.

**Results:**

We demonstrate that siRNA delivery and subsequent silencing of T cell specific genes is substantially increased, if murine T cells were activated prior siRNA transfection. Silencing of ZAP70, p56Lck as well as PLC-γ1 protein expression resulted in impaired function of T cells *in vitro*. Furthermore, delayed type hypersensitivity (DTH) was ameliorated *in vivo *after adoptive transfer of ZAP70-silenced T cells.

**Coclusion:**

The combination of RNAi-mediated gene silencing and adoptive transfer of gene-silenced T cells, thus, may allow the identification and analysis of T cell-specific targets for therapeutic intervention. Additionally, this model system may represent an alternative to conventional time consuming and cost intensive gene targeting approaches.

## Background

RNAi is a naturally occurring mechanism of gene regulation conserved in plants and mammalian cells. The mechanism of RNAi was identified and characterized almost a decade ago in the nematode worm *Caenorhabditis elegans *[1]. It relies on double-stranded (ds), small interfering RNAs (siRNAs) of 21–23 nucleotides that induce sequence-specific gene silencing in mammalian cells [2]. siRNAs derive from long dsRNAs that trigger the RNAi machinery and are cleaved by the RNaseIII-like enzyme Dicer [3]. siRNAs are incorporated into the RNA-induced silencing complex (RISC) that comprises helicase, exonuclease, and homology-searching domains. The siRNA duplex is unwound and the antisense strand mediates mRNA recognition and thereby, mRNA degradation and subsequent gene silencing. Over the last few years, RNAi has evolved as a new technology for specific gene knockdown that allows analysis of gene function *in vitro *and *in vivo *and the identification of new molecular targets associated with disease, such as cancer and inflammation [4-7].

T cells constitute one of the major components of the adaptive, cellular immune system. They play a pivotal role in the onset, balance and maintenance of immune responses as well as autoimmunity. Adaptive immune responses are initiated by interactions of T cells with antigen-presenting cells (APC), such as dendritic cells, in the secondary lymphoid organs. T cells become activated and develop into effector cells by a series of activation signals consisting of i) antigen presentation by APC via peptide-major histocompatibility (MHC) complexes that stimulate the T-cell receptor (TCR, [6]), ii) costimulation by B7 family members that bind to CD28 expressed on T cells [7]. Upon engagement of the TCR, a signaling cascade inside the cell is initiated that leads to activation of the T cell. The Src family of protein tyrosine kinases Fyn and Lck become activated and phosphorylate the cytoplasmic domains of the CD3 complex which then interact with the zeta-chain-associated protein kinase 70 (ZAP70). ZAP70 is subsequently phosphorylated by p56Lck and Fyn and stimulates downstream signaling events leading to the recruitment and activation of a number of other signaling proteins, such as phospholipase C gamma (PLC-γ;[8,9].

Before the discovery of RNAi, studies of gene function mainly relied on knockout strains, protein overexpression and protein-protein interaction assays. However, the knockout of genes results in a complete lack of gene ontology and can cause embryonic lethality or induce compensatory mechanisms. Protein overexpression may result in non physiological conditions and thereby lead to incorrect interpretation of gene function. Whereas successful RNAi in human T cells has been documented in literature [10,11], primary murine T cells are difficult to transfect and therefore published data desribing an efficient protein knockdown in mouse T cells are scarce [12,13].

Here, we report a systematic analysis of RNAi-mediated gene modulation for model target genes in primary murine T cells. We investigated the transfection of siRNA molecules into primary murine T cells *in vitro *and tested such gene-silenced T cells in an adoptive cell transfer model of DTH based on Ovalbumin (OVA) TCR-transgenic T cells *in vivo*. Efficient siRNA delivery and subsequent silencing of ZAP70 protein expression strongly depended on T cell activation. ZAP70-silenced T cells displayed impaired function *in vitro *as well as *in vivo *by inducing less DTH than cells transfected with control siRNA. The combination of RNAi in murine T cells and the *in vivo *analysis of gene-silenced T cells by adoptive transfer may represent a powerful technique to identify and validate novel signaling pathways and inflammation-relevant target genes in T cell biology.

## Methods

### Mice

Eight-12 weeks old BALB/c mice were purchased from Charles River Laboratories (Sulzfeld, Germany). Age- and sex-matched DO11.10 mice were kindly provided by C. Doebis (Deutsches Rheumaforschungszentrum, Berlin, Germany). T cells of DO11.10 mice express a transgenic TCR recognizing OVA_323–339 _peptide bound to I-A^d ^[14]. All animal experiments were performed in accordance with institutional, state, and federal guidelines.

### Generation of T cells and Th1 cells

T cells of BALB/c mice were isolated from splenocytes by immunomagnetic bead purification using the mouse Pan T cell isolation kit according to the manufacturer's instructions (Miltenyi Biotec, Bergisch Gladbach, Germany).

Th1 cells were generated *in vitro *from lymph node cells and splenocytes of DO11.10 mice. Single cell suspensions of splenocytes and lymph node cells were prepared and erythrocytes lysed with 0.83% NH_4_Cl. Cell concentration was adjusted to 2 × 10^6 ^cells/ml and lymph node cells were co-cultured with splenocytes at a ratio of 1:2.5 in 6-well plates. Cells were grown in VLE-RPMI 1640 medium with stable glutamine (Biochrom, Berlin, Germany) containing 10% heat-inactivated FCS, 50 μM β-mercaptoethanol (Sigma, Germany), 100 U/ml penicillin and 100 μg/ml streptomycin sulfate (Invitrogen, Karlsruhe, Germany). Recombinant mouse IFNγ (20 ng/ml), IL-12 (10 ng/ml, both R&D systems) and OVA_323–339 _peptide (1 μg/ml; synthesized at the Department of Biochemistry, Humboldt University, Berlin, Germany) were added to the culture. After 3 days of culture, cells were used for transfection with siRNA and cultured for another 72 h to allow for knockdown of mRNA and protein expression. 72 h after transfection, cells were used for adoptive cell transfer.

### Restimulation

Transfected BALB/c T cells or Th1 cells were restimulated either with plate-bound anti-mouse CD3ε antibody (10 μg/ml; clone 145-2C11, BD Biosciences, Heidelberg, Germany) or with PMA (25 ng/ml, Sigma, Germany) and ionomycin (1 μg/ml, Sigma, Germany). Supernatant was removed 24 h later and analyzed for the secretion of the cytokines interleukin-2 (IL-2) and interferon gamma (IFN-γ) by ELISA (eBioscience, San Diego, USA). For ^3^H-thymidine incorporation cells were pulsed with 0.5 μCi ^3^H-thymidine/well (Amersham Biosciences, GE Healthcare, Munich, Germany) at day 3 of culture. Cells were harvested 6 hours later and ^3^H-thymidine incorporation was measured in a Microbeta Counter (Wallac, Turku, Finland).

### Transfection

After 3 days, 2 × 10^7 ^Th1 cells of DO11.10 mice and/or anti-mouse CD3-stimulated T cells or lymph node cells of BALB/c mice were transfected by nucleofection using the mouse T cell Nucleofector kit and the corresponding transfection programme for murine T cells from AMAXA (Cologne, Germany). Cells were transfected with functional, non-targeting control siRNA (siControl, non-targeting siRNA pool, Dharmacon, Lafayette, USA), a siRNA pool consisting of four siRNAs specific for ZAP710, Lck or PLC-γ1 (siGENOME SMART pool, Dharmacon, Lafayette, USA) or three individual ZAP70-specific Stealth™ siRNAs (Invitrogen, Karlsruhe, Germany). The targeted sequence of the ZAP70-specific stealth™ siRNAs are as follows: stealth™ siRNA #1: 5'UCUCGUACACACUUGUGUCCAUGGG3', stealth™ siRNA #2: 5'AAUUAGUCCAUCGCGCUUCAGCUUC3', stealth™ siRNA #3: 5' UUGUCUGUCGAUGACGCUAAGGCUG3'. Briefly, cells were transfected with different amounts of siRNA and cultured in complete medium and 12-well plates for up to 72 h for subsequent analysis or adoptive cell transfer. Transfection efficiency for GFP plasmid DNA yielded 30% of GFP-positive cells under the applied conditions The transfection effiency with siRNAs was 70–80% determined with fluorescently-labeled siRNAs. Viability of murine T cells transfected by nucleofection was 40–50%.

### RNA isolation, cDNA synthesis and RT-PCR

For the detection of mRNA knockdown by RT-PCR 1 × 10^6 ^transfected cells were lysed with Nucleic Acid Purification Lysis solution (Applied Biosystems, Darmstadt, Germany) and mRNA was isolated using a ABI Prism™ 6100 Nucleic Acid PrepStation (Applied Biosystems, Darmstadt, Germany). cDNA synthesis of RNA samples was performed with TaqMan^® ^Reverse Transcription Reagents (Applied Biosystems, Darmstadt, Germany). Samples were incubated for 10 min at room temperature, 30 min at 48°C and 5 min at 95°C. Subsequent RT-PCR was performed in triplicates for each gene using TaqMan^® ^Gene Expression Assays specific for Lck (Mm 00802897_m1), PLC-γ1 (Mm 01247275_m1), ZAP70 (Mm00494255_m1) and β-actin (Mm00607939_s1, all Applied Biosystems, Darmstadt, Germany) and following the manufacturer's protocol The reactions were run on the GeneAmp^® ^PCR System 9700 thermal cycler with the following amplification program: 50°C for 2 min, 95°C for 10 min, followed by 40 cycles of 95°C for 15 s, 60°C for 1 min. Data was analyzed using Sequence Detection Systems Software v2.2.1 (Applied Biosystems, Darmstadt, Germany) and the gene of interest was normalized to the corresponding β-actin results.

### Protein isolation and Western blot analysis

Protein expression of siRNA-transfected cells was analyzed by Western blot analysis. At least 2 × 10^6 ^cells were lysed in lysis buffer (50 mM Tris-HCl pH 7.5, 150 mM NaCl, 2 mM EGTA, 1 mM NAF, 1% Triton X-100, 1 mM sodium orthovanadate + complete protease inhibitor cocktail tablets (Roche, Mannheim, Germany)). 5 μg of protein lysate determined by BCA Protein Assay Kit (Pierce, Bonn, Germany) and were separated on 10% SDS-PAGE gels and blotted onto PVDF membranes (Biorad, Munich, Germany). Proteins were identified using anti-Lck, (clone 3A5, Millipore, Schwalbach, Germany), anti-PLC-γ1 (clone D-7-3, Millipore, Schwalbach, Germany), anti-ZAP70 (clone 4H386, USBiological, Massachusetts, USA) and anti-β-actin (clone AC-74, Sigma-Aldrich, Schnelldorf, Germany) antibodies. As secondary antibody HRP-conjugated IgG sheep anti-mouse polyclonal serum (GE Healthcare, Munich, Germany) was used and developed with ECL reagents (ECL kit, Amersham Biosciences, GE Healthcare, UK).

### Th1-mediated DTH model

The Th1-mediated DTH model was performed as described before [15] with minor modifications. Briefly, a total of 1 × 10^6 ^transfected Th1 cells generated from DO11.10 mice were injected intravenously into the tail veins of naïve BALB/c mice. Adoptively transferred cells were either mock transfected, transfected with a control siRNA pool or with ZAP70-specific siRNA. 24 h after adoptive cell transfer, the DTH response was induced by subcutaneous injection of 250 ng OVA_323–339 _peptide together with incomplete Freund's adjuvant (IFA) into the right footpad. As control, the left footpad was injected s.c. with PBS/IFA. 24 h after antigen injection, footpad thickness was determined as parameter for the inflammatory response using a specialized micrometer (Bayer Schering Pharma AG, Berlin). Experimental research on animals followed internationally recognized guidelines and was approved by government authorities in Berlin.

### Statistics

Data are presented as mean ± SD. Significance was determined by Mann-Whitney U test. Differences were considered statistically significant with p ≤ 0.05 and highly significant with p ≤ 0.01.

## Results

### Stimulation of murine T cells is required to reduce ZAP70 protein expression via RNAi

**Figure 1:**
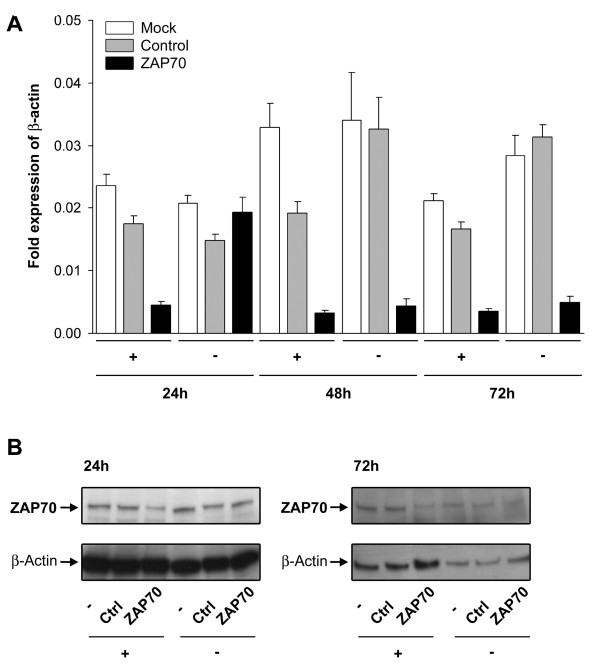
**Stimulation is required to silence ZAP70 expression in T cells**. Murine T cells were stimulated ("+", anti-mouse CD3ε) or left untreated ("-"). 24 h later, cells were transfected with 0.9 μM ZAP70-specific siRNA pool by nucleofection. Cell lysates were prepared 24, 48 and 72 h after transfection and ZAP70 expression was analyzed by RT-PCR (A) and Western blot analysis (B). As control, cells were mock ("-", no siRNA added) transfected () or cells were transfected with a control siRNA pool (Ctrl). (A) RT-PCR of ZAP70 mRNA expression determined as fold expression of the house keeping gene β-actin. (B) Western blot analysis of ZAP70 was detected by ZAP70-specific antibody. β-actin was used as loading control. Shown are the mean values of triplicates of a representative experiments of four independent experiments.

**Figure 2:**
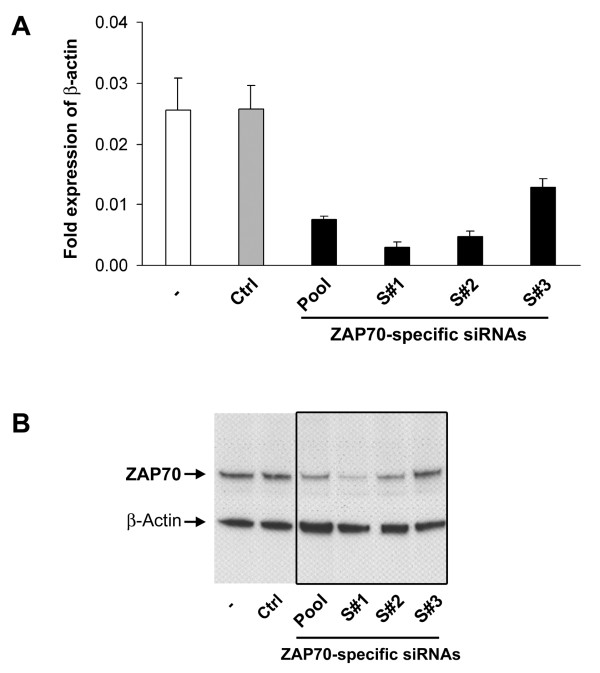
**Modified siRNAs allow efficient silencing of ZAP70 silencing mRNA and protein**. Stimulated murine T cells were transfected with 0.9 μM ZAP70-specific siRNA pool (Pool) or three different stealth™ siRNAs (S#1, S#2, and S#3) by using nucleofection. Cell lysates were prepared 24 and 48 h after transfection and ZAP70 expression was analyzed by RT-PCR (A) and Western blot analysis (B). As control cells were mock transfected ("-", no siRNA added), or transfected with a control siRNA pool (Ctrl). (A) RT-PCR of ZAP70 mRNA expression in transfected T cells determined 24 h after nucleofection as fold expression of the house keeping gene β-actin. (B) Western blot analysis of transfected T cells 48 h after nucleofection with siRNAs indicated in (A). Protein knockdown of ZAP70 was detected by ZAP70-specific antibody. β-actin was used as loading control. Shown are the mean values of triplicates of a representative experiments of two independent experiments.

To investigate whether T cell activation improves transfection efficiency and thereby RNAi-mediated knockdown of ZAP70 in murine T cells, T cells from Balb/c mice were stimulated with anti-mouse CD3ε-specific antibody or left untreated for 24 h. Stimulated and unstimulated cells were transfected with control siRNA or ZAP70-specific siRNA pool by nucleofection and ZAP70 silencing was determined in the cell lysates by RT-PCR and Western blot analysis (Fig. 1). In the unstimulated T cells, mRNA expression of ZAP70 was reduced 48 h and 72 h after nucleofection. Reduced quantities of ZAP70-specific mRNA, however, did not translate into a knockdown of ZAP70 protein (Fig. 1B, note the stronger β-actin signal in the unstimulated ZAP70 probe at 72 hours). In contrast to the unstimulated cells, mRNA and protein silencing of ZAP70 was already detectable in the stimulated cell fraction 24 h after transfection and this was maintained until 72 hours post transfection. The observed correlation of T cell activation and efficient protein knockdown in the case of ZAP70 gene expression, was extended by RNAi studies employing p56Lck and PLC-γ1 as model targets. Here again, stimulation of murine T cells was a prerequisite for successful RNAi-mediated gene silencing (Fig. 2 and data not shown). Hence, T cell receptor-triggered activation seems to be an important prerequisite for efficient RNAi-mediated modulation of protein expression in murine T cells.

### Modified siRNAs allow efficient silencing of ZAP70

Next, we tested the efficiency of unmodified and chemically modified ZAP70-specific siRNAs to induce ZAP70 RNA and protein knockdown. Stimulated T cells were transfected with the previously used ZAP70-specific siRNA pool or three individual stealth™ siRNAs. ZAP70 knockdown was analyzed in the cell lysates by RT-PCR and Western blot 24 and 48 h after nucleofection (Fig. 2). As in previous experiments, the siRNA pool efficiently silenced ZAP70 mRNA and protein expression. Stealth™ siRNA #1 was potent in reducing ZAP70 mRNA and protein expression, whereas stealth™ siRNA # 2 and 3 were less potent as indicated by the RT-PCR and Western blot results. These results demonstrate that our initial findings employing pools of siRNAs can be reproduced with mono-specific, chemically modified siRNAs.

### RNAi is applicable for signaling proteins involved in T cell activation

**Figure 3:**
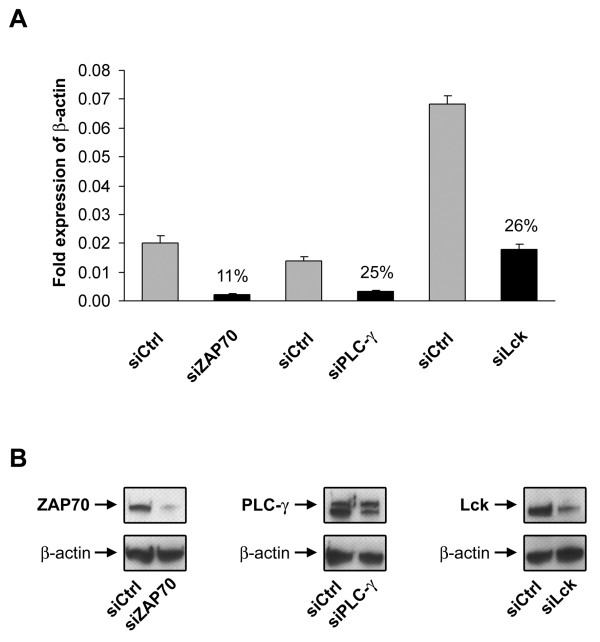
**RNAi is applicable for signaling proteins involved in T cell activation**. Stimulated murine T cells were transfected with 0.9 μM ZAP70-, PLC-γ- or Lck-specific siRNA pool by using nucleofection. Cell lysates were prepared 24 and 48 h after transfection and mRNA and protein expression were analyzed by RT-PCR (A) and Western blot analysis (B). As control, cells were transfected with a control siRNA pool (Ctrl). (A) RT-PCR results of transfected T cells determined 24 h after nucleofection with Control ("siCtrl"), ZAP70 ("siZAP70"), PLC-γ ("siPLC-γ1") and Lck ("siLck") specific siRNAs. (B) Western blot analysis of transfected T cells 48 h after nucleofection. Protein knockdown of ZAP70, PLC-γ and Lck was detected by specific antibodies. β-actin was used as loading control. Shown are the mean values of triplicates of a representative experiments of two independent experiments.

After successful silencing of ZAP70 mRNA and protein expression in murine T cells, we tested RNAi to modulate protein expression of additional, again well-characterized, key molecules involved in T cell signaling. To this end we selected p56Lck and PLC-γ1 for further analysis. Mouse T cells were activated and subsequently transfected with a siRNA control pool or siRNA pools specific for p56Lck, PLC-γ, or ZAP70. Transfection with specific siRNAs strongly reduced ZAP70 mRNA expression in activated T cells to background levels, while transfection with both p56Lck and PLC-γ siRNA pools reduced mRNA expression by 75% (Fig. 3). The results of Western blot analysis correlated well with the RT-PCR results: ZAP70 protein expression under the experimental conditions was substantially reduced, whereas p56Lck and PLC-γ proteins were still present in the transfected T cells although at reduced levels as detected in Western blot.

### RNAi of T cell-specific signaling molecules alters murine T cell function *in vitro*

The effect of RNAi-induced p56Lck, PLC-γ and ZAP70 protein knockdown on T cell function was further investigated *in vitro *quantifying the proliferation and cytokine release of siRNA-treated T cells. Previously activated T cells, transfected with p56Lck, PLC-γ1 or ZAP70 siRNAs, were incubated for 3 days and then restimulated by TCR-specific (anti-CD3 antibody) or TCR-independent (PMA and ionomycin) treatment. IL-2 and IFN-γ secretion and proliferation were determined by ELISA and ^3^H-thymidine incorporation, respectively (Table 1). Both, cytokine production and cell proliferation, were clearly reduced in T cells transfected with p56Lck-, PLC-γ1 or ZAP70-specific siRNAs, when the T cells were activated via the TCR. RNAi of the tested genes specifically interfered with TCR-dependent pathways and does not exert its function via non-specific, e.g. toxic transfection-related mechanisms, since all transfected T cells equally well responded to PMA and ionomycin stimulation. Hence, using the described experimental setup, i.e. activation of T cells and nucleofection, RNAi allows specific loss-of-function studies in primary murine T cells *in vitro*.

**Table 1:** Proliferation, IL-2 and IFN-γ production of stimulated and siRNA-transfected BALB/c T cells.

	**siRNA**	**IL-2 [pg/ml]**	**IFN-γ [pg/ml]**	**Proliferation [CPM]**
**αCD3**				
	Ctrl	1417 ± 144	965 ± 105	4106 ± 433
	Lck	443 ± 93	357 ± 50	540 ± 220
	PLC-γ	195 ± 28	146 ± 26	236 ± 19
	ZAP70	400 ± 60	467 ± 149	568 ± 69

**PMA + Ionomycin**				
	Ctrl	1980 ± 2	5067 ± 346	19919 ± 787
	Lck	2167 ± 0	4158 ± 135	16640 ± 954
	PLC-γ	1676 ± 28	2939 ± 88	11285 ± 658
	ZAP70	1991 ± 2	4603 ± 277	15987 ± 1906

T cells were transfected with 0.9 μM Lck-, PLC-γ- and ZAP70-specific siRNA pool, ZAP70-specific stealth™ siRNA 1 and with control siRNA pool (Ctrl). 72 h after nucleofection, functionality of transfected cells was tested by restimulation with anti-mouse CD3 antibody or PMA and ionomycin in vitro. IL-2 and IFN-γ production was determined in the supernatant 24 h after restimulation by ELISA. Means of duplicate values are shown. Proliferation of T cells was detected by ^3^H-thymidine incorporation 96 h after restimulation. Values of ^3^H-thymidine incorporation are shown in means of triplicates.

### RNAi of ZAP70 alters murine T cell function *in vivo*

**Figure 4:**
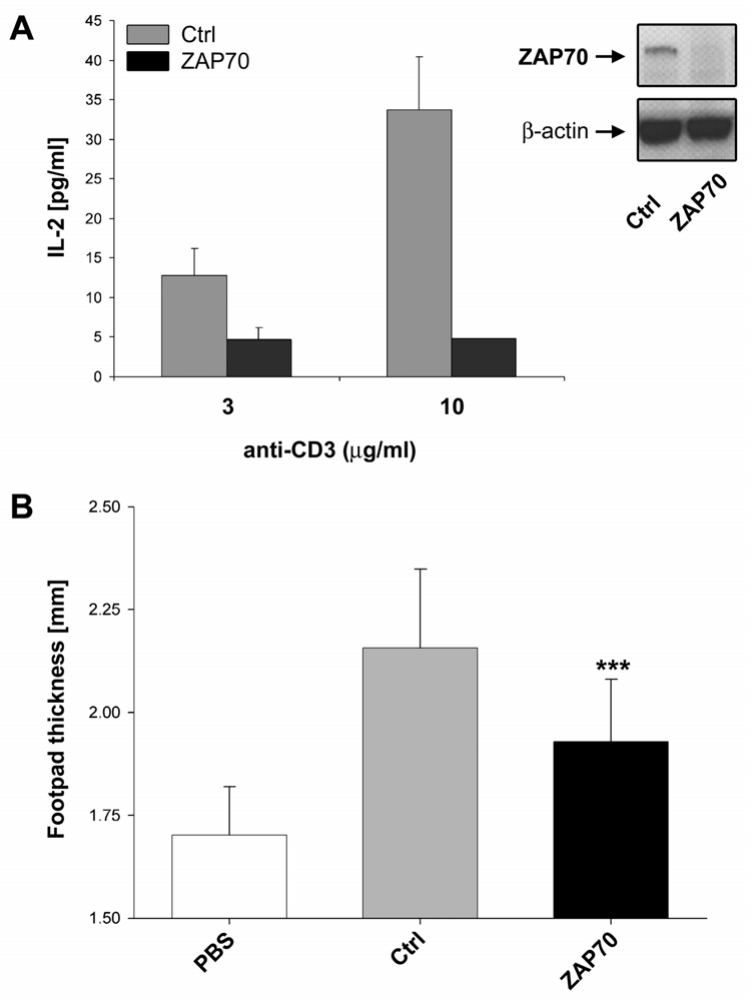
**RNAi-mediated knockdown of ZAP70 affects T cell function *in vitro *and *in vivo***. DO11.10 T cells were either mock transfected, transfected with a control siRNA pool (Ctrl), or transfected with 0.9 μM ZAP70-specific stealth™ siRNA #1 (ZAP70). 72 h after nucleofection, functionality of transfected cells was tested by restimulation (α-CD3) *in vitro *or by adoptive cell transfer into naïve recipient mice *in vivo*. (A) 1 × 10^6 ^DO11.10 T cells were restimulated with 3 or 10 μg/ml anti-mouse CD3 antibody. IL-2 production was determined in the supernatants by ELISA. Means of duplicate values are shown.(B) 1 × 10^6 ^transfected Th1 cells were adoptively transferred via i.v. injection into Balb/c mice. Cells were either mock transfected, transfected with a control siRNA pool or with ZAP70-specific siRNA. 24 h after adoptive cell transfer, DTH response in the footpad was induced by s.c. injection of OVA peptide/IFA in recipients of cells transfected with control or ZAP70-specific siRNA. As control, recipient mice having obtained mock transfected cells were challenged with PBS/IFA. Footpad thickness was determined 24 h after antigen exposure. (*** p ≤ 0.001). Shown is the mean value of 20 mice in total (per group) analyzed in three independent experiments.

In order to analyze murine T cells *in vivo*, we used T cells from DO11.10 TCR-transgenic T cells, which allows the induction and tracing of an immune response with defined antigen specificity *in vitro *and *in vivo*. Since in our hands the RNAi-mediated protein knockdown was most efficient for the T cell-specific ZAP70 as model gene, activated DO11.10 T cells transfected with ZAP70 specific siRNAs were first functionally analyzed by *in vitro *restimulation. Transfection of DO11.10 T cells with ZAP70-specific siRNAs, as shown before for non TCR-transgenic murine T cells (Table 1), resulted in substantially reduced IL-2 secretion after CD3-mediated stimulation (Fig. 4A). Subsequently, ZAP70-silenced T cells were further investigated in an adoptive cell transfer model of DTH (Fig. 4B). Adoptive transfer of transfected DO11.10 T cells into Balb/c mice, elicited a significantly (p ≤ 0.001) reduced DTH response in the recipient mice compared to T cells transfected with control siRNA. Our results show that RNAi-mediated modulation of T cell function can also be exploited in inflammatory model systems *in vivo*.

## Discussion

The present study describes the application of RNAi technology in primary murine T cells and for the first time its successful application in an adoptive cell transfer model of DTH *in vivo*.

In our hands, activation of murine T cells was a prerequisite for successful RNAi-mediated knockdown. This has not been described in detail before (Choi et al., 2006; Chung et al., 2007) and may be specific for the nucleofection transfection technology we employed in combination with our cells of interest – murine T cells. For optimal RNAi-induced protein knockdown several factors like cellular uptake, half-life of the target mRNA (and the protein), and the structure and accessibility of the target mRNA need to be considered [16-18]. Possibly, activation of murine T cells improves the accessibility of the target mRNA and/or structure as well as transfection efficiency. Using GFP reporter constructs (data not shown), we could demonstrate that in activated and transfected murine T cells a higher percentage of the T cells was GFP-positive when compared to naïve, unstimulated murine T cells.

Our data demonstrate that knockdown efficiency can differ between the genes of interest and are in line with data describing that sequence, structure, half-life and turnover rate of the target mRNA need to be considered for successful RNAi-based modulation of protein expression [4,5,19]. The investigations presented in this study intentionally focussed on known model proteins being critical for TCR-mediated signaling events during T cell activation in order to have a clear phenotypic readout, e.g. reduced cytokine secretion, in the silenced cells. We clearly show that an efficient siRNA-mediated knockdown of p56Lck, PLC-γ1 and ZAP70 is possible in activated murine T cells with the consequence that the siRNA-treated T cells exhibited an altered functional phenotype regarding cytokine secretion and proliferation. Having optimized the experimental conditions for siRNA delivery into murine T cells, our results may be extended to other relevant molecules expressed in T cells in future investigations.

Recently, RNAi technology also facilitated more detailed investigations on the role of kinases in TCR-mediated signaling using *in vitro *activated T cells or T cell lines [20-22]. Here, we describe the impact of a kinase knockdown (ZAP70) *in vivo *by an adoptive cell transfer model of DTH. This adoptive cell transfer model offers the unique advantage to evaluate the role of target genes in its corresponding *in vivo *environment of inflammation. Loss-of-function studies by RNAi and adoptive cell transfer may, thus, represent a new method to study direct correlations between gene expression and the function of the respective protein in an inflammatory response. Murine *in vivo *models rely mainly on transgenic and knockout strains. However, gene targeting in mice can, on one hand, cause embryonic lethality or on the other hand, in the case of transgenic expression, lead to non-physiological protein expression. Although the number of elegant inducible knockout strains is constantly increasing, experiments employing these mouse strains are time consuming and cost-intensive. Here, we show that under defined experimental conditions, i.e. pre-activation of the T cells, RNAi technology represents a powerful tool to validate T cell specific genes in murine T cells, which can subsequently be used in established *in vivo *animal models to better understand the regulation and contribution of inflammation-relevant genes.

## Competing interests

The authors declare that they have no competing interests.

## Authors' contributions

TCG, LN performed the experiments and were involved in drafting the manuscript (TCG). CM contributed in analysis and interpretation of the data, KA, UZ contributed to conception and design of the data and AvB has been involved in designing the studies and drafting/revising the manuscript.
